# Process Optimization on Micro-Aeration Supply for High Production Yield of 2,3-Butanediol from Maltodextrin by Metabolically-Engineered *Klebsiella oxytoca*

**DOI:** 10.1371/journal.pone.0161503

**Published:** 2016-09-07

**Authors:** Sitha Chan, Sirima Suvarnakuta Jantama, Sunthorn Kanchanatawee, Kaemwich Jantama

**Affiliations:** 1 Metabolic Engineering Research Unit, School of Biotechnology, Institute of Agricultural Technology, Suranaree University of Technology, 111 University Ave., Suranaree Sub-district, Muang District, Nakhon Ratchasima, 30000, Thailand; 2 Division of Biopharmacy, Faculty of Pharmaceutical Sciences, Ubon Ratchathani University, Warinchamrap, Ubon Ratchathani, 34190, Thailand; University of Huddersfield, UNITED KINGDOM

## Abstract

An optimization process with a cheap and abundant substrate is considered one of the factors affecting the price of the production of economical 2,3-Butanediol (2,3-BD). A combination of the conventional method and response surface methodology (RSM) was applied in this study. The optimized levels of pH, aeration rate, agitation speed, and substrate concentration (maltodextrin) were investigated to determine the cost-effectiveness of fermentative 2,3-BD production by metabolically-engineered *Klebsiella oxytoca* KMS005. Results revealed that pH, aeration rate, agitation speed, and maltodextrin concentration at levels of 6.0, 0.8 vvm, 400 rpm, and 150 g/L respectively were the optimal conditions. RSM also indicated that the agitation speed was the most influential parameter when either agitation and aeration interaction or agitation and substrate concentration interaction played important roles for 2,3-BD production by the strain from maltodextrin. Under interim fed-batch fermentation, 2,3-BD concentration, yield, and productivity were obtained at 88.1±0.2 g/L, 0.412±0.001 g/g, and 1.13±0.01 g/L/h respectively within 78 h.

## Introduction

Bio-refinery systems that integrate bio-mass conversion processes and are equipped to produce fuel, power, and bio-based chemicals from renewable resources are the focus of worldwide development due to concerns about scarce crude oil reserves, gradual increases in price, and environmental pollution [1, 2]. 2,3-Butanediol (2,3-BD) is one example of bulk chemicals produced by fermentation that has raised much interest. An extensive application for 2,3-BD occurs in various fields, such as fuel, chemical industry, food industry, softening agents, explosives, plasticizers, and pharmaceutical agents. Furthermore, dehydration of 2,3-BD can be used in industrial solvents, such as methyl ethyl ketone [3].

There are several micro-organisms, including *Klebsiella pneumoniae*, *K*. *oxytoca*, *Bacillus polymyxa*, *Serratia marcescens*, capable of naturally producing 2,3-BD [4]. Among these species, *Klebsiella* spp. were comprehensively studied for fermentative 2,3-BD production [5–9]. For example, Yu and Saddle [5] obtained a very high concentration of 2,3-BD at the level of 113 g/L using a fed-batch operation by *K*. *pneumonia*. Ma et al. [6] also utilized *K*. *pneumonia* SDM isolated from soil for 2,3-BD production with very high productivity from glucose. However, *K*. *oxytoca* has an advantage at the purification step over *K*. *pneumoniae* owing to less formation of capsular polysaccharide during 2,3-BD production [10]. Therefore, this may make *K*. *pneumoniae* unsuitable for 2,3-BD production on a large scale.

The conventional optimization method and response surface methodology (RSM) were employed for the fermentative production of 2,3-BD. These strategies were mostly applied in flask experiments prior to bio-reactors leading to medium optimization. However, only a few studies reported about process optimization of physiological parameters affecting 2,3-BD, such as pH, aeration, and agitation [11]. Some attempted to establish proper oxygen supply strategies to ensure efficient 2,3-BD production. Strategies to control parameters, including oxygen transfer rate (OTR), volumetric oxygen transfer coefficient (K_L_a), oxygen uptake rate (OUR), and respiratory quotient (RQ), were applied for 2,3-BD fermentation. However, the application of those strategies is restricted as these parameters are not easily controlled [3].

Most previously-published works reported the use of complex and rich nutrients for the promotion of microbial growth and improvement of 2,3-BD production [6–9; 11–17]. It is expected that high production costs related to medium preparation, downstream processing, and waste disposal have not substantially met criteria for process economy [18]. Robust micro-organisms, the use of inexpensive substrates and media, and simple downstream processes were taken into account to ensure the economic feasibility of 2,3-BD production [19]. In addition, the development of a simple fermentative production process while utilizing cheap agricultural substrates is vital to obtain high concentrations and yields of 2,3-BD. Maltodextrin, a product of partially hydrolyzed corn or cassava starch, is a cheap and abundant carbon source that may be fermented to 2,3-BD due to its highly pure substrate, accessible digestion, and rapid absorption. There is only one publication of the use of maltodextrin for ethanol production [20]. Therefore, the use of a low-cost medium without supplementation of any complex, rich, and expensive nutrients with a cheap substrate such as maltodextrin is an attractive consideration for the economic production of 2,3-BD.

In this study, optimized parameters affecting 2,3-BD production were investigated with a recently published *K*. *oxytoca* KMS005 (Δ*adhE*Δ*ack-pta*Δ*ldhA*) strain [21] using maltodextrin as the sole carbon source supplemented in a mineral salt medium. By the use of conventional optimization coupled with RSM, the optimized levels of four parameters (pH, aeration rate, agitation speed, and maltodextrin concentration) were precisely indicated. A single stage fermentation was established and optimized using maltodextrin to produce 2,3-BD with a high production yield in batch and interim fed-batch fermentation. At 78 h, the concentration of 2,3-BD was obtained at the level of 88.1 g/L (0.412 g/g maltodextrin used). Interestingly, the low cost of fermentative 2,3-BD production calculated at $2.04/kg based on this study may be effective in competition with chemical-based 2,3-BD production.

## Material and Methods

### Microorganism and media

Metabolically-engineered *K*. *oxytoca*, KMS005 (Δ*adhE-*Δ*ack-pta*Δ*ldhA*) was previously produced [21]. Maltodextrin was purchased in Nakhon Ratchasima, Thailand. The percentage of solid content in maltodextrin is approximately 84% (w/w) and its dextrose equivalent (DE) is 6.4. A simple mineral salt medium (4 g/L of salts), AM1 [22] was used as a fermentative medium throughout this study. Luria-Bertani (LB) agar was used for maintaining bacterial cultures.

### Culture conditions

For seed preparation, KMS005 was cultured on a Luria-Bertani (LB) agar. The plate was incubated at 37°C for 24 h. A full single loop of fresh colonies was inoculated into 250 mL Erlenmeyer flasks containing 60 mL LB medium. The inoculum was incubated at 37°C and 200 rpm for 16 h. The seed culture was inoculated in AM1 medium supplemented with maltodextrin at the concentration equivalent to 0.033 g/L dry cell weight (OD_550_ = 0.1). Fermentation experiments were carried out at 37°C in a 2 L scale bio-reactor with a working volume of 1 L. The fermentation broth was supplied with sterile air at desired flow rates and controlled at desired agitation speeds. The pH of the medium was constantly maintained by the automatic addition of 3.0 M KOH. The percentage of dissolved oxygen was also measured by dissolved oxygen (DO) probe.

### Process optimization of 2,3-BD production

The levels of the parameters affecting 2,3-BD, pH, aeration rate, agitation speed, and maltodextrin concentration, were initially screened using a ‘one variable at a time’ strategy prior to subjection to RSM. The parameters were: pH (5.0, 5.5, 6.0, 6.5 and 7.0); aeration rates (0.1, 0.5, 0.8, 1.0 and 1.2 vvm); agitation rates (200, 300, 400 and 500 rpm); maltodextrin concentrations (5, 10, 15, 20 and 25% (w/v)). Experiments were repeated in triplicate. Data were analyzed by the SPSS program (version 15.0). Comparisons between means were carried out using a Duncan’s new multiple range test at P < 0.05.

RSM based on the Box-Behnken design was employed to determine optimized levels of aeration rate, agitation speed, and maltodextrin concentration for 2,3-BD production by the KMS005 strain. Design expert version 8.0 was used to program the Box-Behnken design to maximize the response of 2,3-BD production. All fermentation experiments were performed using a 2 L bio-reactor in which pH was controlled at 6.0 and temperature was at 37°C.

### Batch and fed-batch fermentations

Optimized conditions obtained from RSM were validated under batch conditions prior to being employed under fed-batch conditions. During an interim-feeding fermentation, maltodextrin solution at the concentration of 80% (w/v) was added to the bio-reactor thus maintaining sugar in the broth at concentrations of 45–60 g/L when the residual sugar concentrations were in the range of 30–35 g/L.

### Analytical methods

Two milliliters of culture broth were withdrawn every 6 h to measure the concentrations of cell bio-mass, organic acids, and sugars. Cell mass was estimated from the optimal density at 550 nm (0.033 g/L of dry cell weight of OD_550_ is 0.1) with a spectrophotometer Spekol^®^1500. Sugars, 2,3-BD, and other by-products were determined by the use of high performance liquid chromatography (HPLC) (Agilent, 2009) equipped with UV and refractive detectors with a Bio-Red Aminex HPX-87H ion exclusion column. Sulfuric acid was used as a mobile phase at the concentration of 4 mM. Residual maltodextrin left in the broth was fully hydrolyzed to be glucose units. A Glucoamylase (Siam Victory Chemicals Co., Ltd.) at 500 U was added to the broth. The reaction was incubated for 2 hours at 65°C and 200 rpm prior to filtration. Ten micro-liters of filtered broth were injected for analysis by HPLC. A percentage of dissolved oxygen (DO) in fermentation was measured by dissolved oxygen probe. The probe was mounted in the medium prior to calibration by de-gassing with nitrogen gas. After inoculation, decreasing values of DO as a function of time were recorded.

## Results and Discussion

### Effects of pH

The effects of pH in the range of 5.0 to 7.0 on 2,3-BD production by the KMS005 strain were initially investigated at the aeration rate and agitation speed of 0.5 vvm and 200 rpm respectively, and maltodextrin at the concentration of 100 g/L (equivalent to 93 g/L glucose) was used. As shown in Table 1, 2,3-BD production at the concentration of 31.8±0.3 g/L was maximized with a yield of 0.34±0.01 g/g maltodextrin at pH 6.0. However, there was no significant difference in 2,3-BD production at pH 6.5 in both 2,3-BD and yield compared to the results at pH 6.0. Detectable levels of acetate, lactate, and formate were not observed at pH 6.0. It may be implied that acetate acted as an inducer and may be directed and utilized for 2,3-BD production through the activity of α-acetolactate synthase. Noticeably, an increase in pH over 6.5 resulted in lowering 2,3-BD production in terms of concentration and yield. In addition, the highest levels of by-products, mainly succinate and acetate, were accumulated at pH 7.0. This observation was in agreement with Lee et al. [7] whose study stated that an increased pH led to higher proportions of fermentative metabolites such as acetate, suucinate, and ethanol, resulting in a decreased level of 2,3-BD in *K*. *pneumoniae*. This may be due to an inactivation of α-acetolactate synthase activity at higher levels of pH than 6.0. Stormer [23] also found that a pH above 6.0 caused a sharp decrease in the activity of α-acetolactate synthase in the 2,3-BD producing pathway in *K*. *pneumoniae*, thus diverting the carbon flow to other fermentative pathways. Our findings also demonstrated that lower pH values than 6.0 caused significant reductions in substrate utilization and biomass formation, thus detrimentally affecting 2,3-BD production. This may be explained by inefficient maltodextrin transport at low pH conditions. Pajatsch et al. [24] demonstrated that *Klebsiella* species was able to deliver linear maltodextrins (maltose up to maltoheptaose) through actions of a binding protein-dependent ABC transporter. Pedersen and Carafoli [25] revealed that the prevailing reaction for ATP synthesis via proton motive force (PMF) by ATP synthase was not thermodynamically favored at pH lower than 6.9. Our results clearly showed that maltodextrin was greatly accumulated in the fermentation broth at lower pH values and even uncontrolled pH conditions (Table 1). These results may suggest that the sufficiently high acid concentration caused a collapse of the pH gradient across the cell membrane at pH values less than 6.0, resulting in an impairment of ATP production via PMF. Inefficient ATP production may cause the activity of the ABC transporter for maltodextrin to be ineffective at low pH values. In addition, the KMS005 strain was tested for 2,3-BD production under a non-controlled pH experiment (Table 1). This resulted in the lowest production of 2,3-BD (11.6±1.5 g/L) when pH in the medium was changed from 7.2 to 4.8. This finding confirmed that maltodextrin was not efficiently utilized and not consumed by the strain when pH was gradually lowered in an uncontrolled pH experiment, indicating the highest level of residual sugar accumulating in the fermentation broth. This result did not agree with Biebl et al. [26] who claimed that 2,3-BD concentration and yield were impressively obtained when the pH of the fermentation broth of *K*. *pneumoniae* was not controlled. The pH values of the broth changed continuously from 7.0 to 5.5. Therefore, our findings that contrasted with the work of Biebl et al. [26] may be caused by differences of micro-organism strains, bio-reactor configurations, and operational conditions.

**Table 1:** Fermentation profiles for 2,3-BD production by *K*. *oxytoca* KMS005 at various pH values in AM1 medium containing 100 g/L maltodextrin.

pH	Residual sugar	Max CDW	2,3-BD	Gross yield	Productivity	By-products (g/L)				
	(g/L)	(g/L)	(g/L)	(g/g)^a^	(g/L/h)^a^	Suc^d^	Lac	Eth	For	Ace
uncontrolled	67.1±2.2	1.0±0.1	11.6±1.5^c^^,β^	0.12±0.01^β^	0.16±0.01	ND^b^	ND	ND	ND	1.5±0.1
5	60.1±4.3	2.4±0.1	15.1±1.8^γ^	0.16±0.01^γ^	0.21±0.02	ND	ND	ND	ND	1.4±0.3
5.5	33.0±2.2	2.9±0.1	23.2±1.6^θ^	0.25±0.01^θ^	0.32±0.02	1.8±0.3	ND	ND	ND	1.1±0.1
6	5.3±1.2	3.9±0.1	31.8±0.3^π^	0.34±0.01^π^	0.44±0.01	2.2±0.1	ND	1.9±0.1	ND	ND
6.5	1.0±0.3	4.2±0.1	31.3±1.2^π^	0.33±0.01^π^	0.43±0.02	3.4±0.1	ND	2.2±0.1	ND	1.7±0.1
7	ND	3.5±0.4	26.2±0.6^Ұ^	0.28±0.01^Ұ^	0.36±0.04	8.6±0.4	ND	ND	ND	10.7±0.9

^a^ Gross yield was calculated as product concentration divided by initial total sugar concentration equivalent to 93 g/L of sugar content at 72 h incubation. Productivity was calculated at 72 h

^b^ ND = not detected

^c^ All data represent the averages of two fermentations with standard deviations. Values bearing different Greek symbols such as “β”, “γ”, “θ”, “π”, and “Ұ” are significantly different (P < 0.05)

^d^ Suc: Succinate, Lac: Lactate, Eth: Ethanol, For: Formate, and Ace: Acetate

### Effects of aeration rates

The effects of aeration rates ranging from 0.1 to 1.2 vvm to 2,3-BD production were also investigated to find a suitable level of micro-aerobic conditions to ensure an activation of enzymes involved in 2,3-BD production, such as α-acetolactate synthase and 2,3-BD dehydrogenase [7]. Experiments were performed at fixed parameters of 200 rpm agitation speed, pH 6.0, and 10% (w/v) maltodextrin. As shown in Table 2, higher 2,3-BD concentrations ranging from 32 to 34 g/L and their improved productivities ranging from 0.67 to 0.72 g/L/h were observed as aeration rates varied from 1.0 to 1.2 vvm. The results revealed non-significant differences in 2,3-BD concentrations and yields (p<0.05) between both aeration rates. Thus, the lower aeration rate at 1.0 vvm was sufficient enough to provide preferable conditions for 2,3-BD production by the KMS005 strain. Stanbury and Whitaker [27] suggested that the aeration rate should be in the range of 0.5–1.0 vvm. An aeration rate in this range is maintained at scale-up operations for lower energy consumption and industrially operational availability.

**Table 2:** Fermentation profile for 2,3-BD production by *K*. *oxytoca* KMS005 at various aeration rates in AM1 medium containing 100 g/L maltodextrin.

Aeration	Residual sugar	Max CDW	2,3-BD	Gross yield	Productivity	By-products (g/L)		
(vvm)	(g/L)	(g/L)	(g/L)	(g/g)^a^	(g/L/h)^a^	Suc	Eth	Ace
No air	84.0±3.0	0.6±0.1	1.6±0.1^c^^,β^	0.03±0.01^β^	0.02±0.01	0.8±0.1	0.9±0.1	2.1±0.1
0.1	78.6±1.8	2.2±0.2	6.2±0.3^γ^	0.06±0.01^γ^	0.12±0.01	0.6±0.1	ND^b^	1.0±0.1
0.8	12.7±1.9	4.7±0.2	27.9±0.1^α^	0.30±0.01^α^	0.58±0.01	3.2±0.1	2.2±0.2	0.6±0.1
1	4.5±2.5	4.4±0.2	32.1±1.4^¥^	0.34±0.01^Ұ^	0.67±0.03	2.2±0.2	1.4±0.3	1.1±0.9
1.2	7.9±1.6	4.7±0.1	34.6±2.2^¥^	0.35±0.02^Ұ^	0.72±0.05	1.5±0.2	1.3±0.1	0.2±0.1

^a^ Gross yield was calculated as product concentration divided by initial total sugar concentration (10% (w/v) at 48 h incubation. Productivity was calculated at 48 h.

^b^ ND = not detected.

^c^ All data represent the averages of two fermentations with standard deviations. Values bearing different Greek symbols such as “β”, “γ”, “α”, “¥” and “Ұ” are significantly different (P < 0.05).

It should be noted that the aeration rate had an equivalent effect on 2,3-BD production and cell growth compared to those of pH (Tables 1 and 2). The majority of enzymes involving in 2,3-BD producing pathway is activated under micro-aerobic conditions, and irreversibly inactivated by oxygen under fully aerobic conditions. This was clearly confirmed by our results (Table 2). The concentrations and production yields of 2,3-BD were significantly increased when aeration rates were increased to 1.2 vvm. Voloch et al. [8] suggested that oxygen was less soluble in water than those of carbon substrates was. Thus, the oxygen demand of an industrial fermentation process for 2,3-BD production is normally satisfied by aerating the fermentation broth. Then, the production yield of 2,3-BD can be maximized by adjusting the suitable oxygen supply to limit the respiration. Our results revealed that the 2,3-BD production yields and concentrations improved when increased suitable aeration rates were provided.

Generally, ethanol is usually produced in approximately equimolar amounts with 2,3-BD along with formate, acetate, lactate, and acetoin by *K*. *oxytoca* in the absence of oxygen. However, the KMS005 strain deleted *adhE* and *ldhA* genes encoding enzymes involved in NADH re-oxidation pathways. Two pathways in central metabolism for succinate and 2,3-BD productions are responsible for NADH reoxidation in the KMS005 strain. No lactate was observed but some amounts of ethanol were detected (Table 2). It was likely that other isoenzymes, such as acetaldehyde dehydrogenase 2 (encoded by *mhpF*), ethanol dehydrogenase P (encoded by *adhP*), acetaldehyde dehydrogenase (encoded by *ald*), and 1,3- propanediol oxidoreductase (encoded by *yqhD-1*), were suspected of taking responsibility for ethanol formation under micro-aerobic conditions [9,28]. These genes are usually activated by FNR (fumarate-nitrate reduction regulatory) protein. A high level of active FNR protein appeared to be present during micro-aerobic respiration [29]. Therefore, it is suspected that FNR activity is correlated with the high redox potential (NADH/NAD^+^ ratio) obtained under micro-aerobic growth performed in our study.

Succinate was still detected under micro-aerobic conditions (0.8–1.2 vvm) but KMS005 seemed to not effectively produce 2,3-BD and succinate under non-aerated conditions (Table 2) due to impaired growth. Usually, succinate-producing pathway via phosphoenol pyruvate (PEP) carboxylase (PPC: encoded by *ppc*) and malate dehydrogenase (MDH: encoded by *mdh*) activities are responsible for NADH re-oxidation under anaerobic conditions [30, 31]. The results showed that the KMS005 strain more preferably dissimilated PEP fluxes through 2,3-BD production pathway to conserve free energy and to reduce the ratio of NADH to NAD^+^ respectively. This did not prevent the production of succinate. This situation may be explained by the fact that PEP is also essential to supply precursor metabolites, including amino acids for biomass formation. Then, the requirement of intracellular PEP in KMS005 was still maintained with a small extent of carbon flux to oxaloacetate (OAA). Upon OAA availability, KMS005 strain may produce succinate from OAA via MDH activity to partially maintain the redox balance (NADH/NAD^+^ ratio). Recently, Jantama et al. [21] revealed that the KMS005 strain exhibited a low level of MDH activity during micro-aerobic conditions. The *mdh* expression was kept at very low levels during micro-aerobic conditions by the activity of ArcA (encoded by *arcA*) protein [29]. In addition, it was also demonstrated that the MDH activity was highest based on an availability of OAA under micro-aerobic conditions [32].

On the other hand, succinate accumulation may be involved with an activity of putative FNR protein. In 2,3-BD pathway, three genes encoding for α-acetolactate synthase, α-acetolactate decarboxylase, and acetoin reductase are known to be clustered in one operon called *budABC*. In the operon, FNR binding site appeared at position -6 to facilitate transcription and regulation of the gene cluster during the anaerobic process [9, 33]. At low aeration (0.8 vvm), FNR may down-regulate the expression of *budABC* genes causing the highest production of succinate. By increasing aeration, more oxygen was able to activate α-acetolactate synthase while FNR was down-regulated resulting in a low expression of genes involving succinate-producing pathway, including fumarate dehydrogenase (FDH: encoded by *fdh*). FDH activity functionally converts fumarate to succinate under both anaerobic and micro-aerobic conditions. Hence, the more aeration increased, the more succinate decreased. It may also be concluded that high 2,3-BD production along with some produced levels of succinate and ethanol showed that the ratio of NADH/NAD^+^ was well-balanced to prevent a retarded glycolytic flux caused by the low efficiency of NADH re-oxidation.

### Effects of agitation speeds

**Fig 1:**
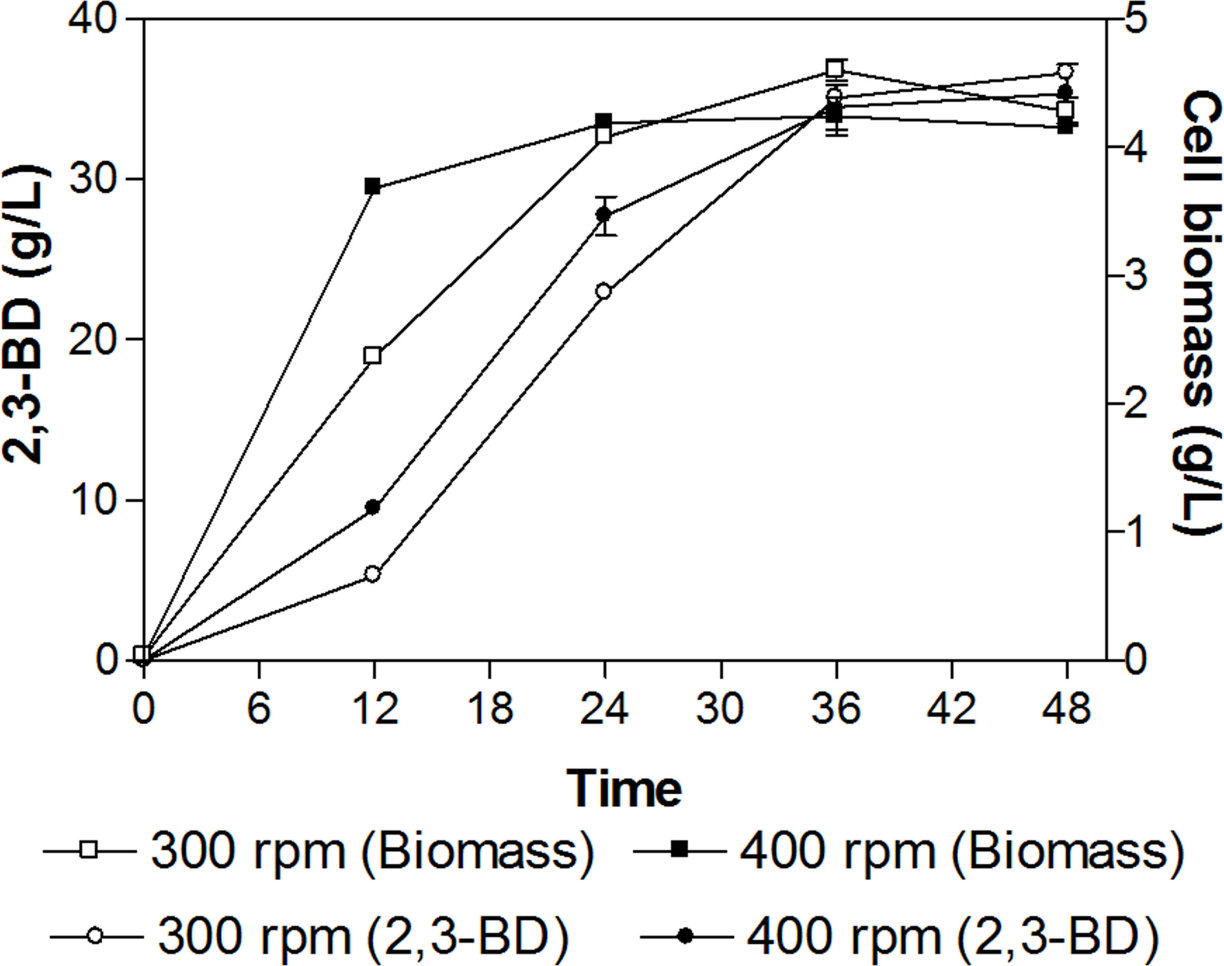
Comparison of cell biomass and 2,3-BD production at 300 and 400 rpm. Aeration, pH, and maltodextrin concentration were fixed at levels of 1.0 vvm, 6.0, and 10% (w/v) respectively.

Agitation speeds were varied from 200 to 500 rpm at the fixed aeration rate of 1.0 vvm, pH 6.0, and maltodextrin concentration of 10% (w/v) to investigate the effects on 2,3-BD production. Our results revealed that the agitation speed at 400 rpm provided a faster cell growth (Fig 1) accompanied with the high 2,3-BD concentration of 35 g/L. The agitation speed at 400 rpm led to faster cell growth but provided a comparable 2,3-BD production than that obtained at the agitation speed of 300 rpm (Fig 1; S1 Table). There was no significant differences in 2,3-BD concentrations and yields between agitation rates of 300 and 400 rpm at 48 h incubation (Table 3). Our findings were supported by Cho et al. [34] who stated that the agitation speed of 400 rpm was the best condition to drive both cell growth and 2,3-BD production using *K*. *oxytoca* species from glucose.

**Table 3:** Fermentation profile for 2,3-BD production by *K*. *oxytoca* KMS005 at various agitation speeds in AM1 medium containing 100 g/L maltodextrin.

Agitation	Residual sugar	Max CDW	2,3-BD	Gross yield	Productivity	By-products (g/L)		
(rpm)	(g/L)	(g/L)	(g/L)	(g/g)^a^	(g/L/h)^a^	Suc	Eth	Ace
200	4.5±2.5	4.4±0.2	32.1±1.4^b^^, β^	0.34±0.01^β^	0.67±0.03	2.2±0.2	1.4±0.3	1.1±0.9
300	1.6±0.1	4.8±0.1	36.6±0.6^π^	0.39±0.01^π^	0.76±0.01	2.2±0.4	0.8±0.1	1.2±0.2
400	1.0±0.1	4.4±0.1	35.4±2.6^π^	0.38±0.02^π^	0.73±0.05	1.4±0.1	<0.1	0.9±0.1
500	31.3±3.1	4.4±0.2	23.7±0.1^¥^	0.25±0.01^¥^	0.49±0.01	<0.1	<0.1	2.6±0.6

^a^ Gross yield was calculated as product concentration divided by initial total sugar concentration (10% (w/v) at 48 h incubation. Productivity was calculated at 48 h.

^b^ All data represent the averages of two fermentations with standard deviations. Values bearing different Greek symbols such as “β”, “π”, and “¥”are significantly different (P < 0.05).

Banks [35] demonstrated that the degree of agitation had a profound effect on the oxygen-transfer efficiency in agitated fermenters. Based on our results, a lower agitation speed at 200 rpm did not only cause a decrease in 2,3-BD production in terms of yield and concentration but also accumulation of high levels of succinate, ethanol, and acetate (Table 3). This indicated that the conditions at agitation rates of 200 to 300 rpm favored succinate and ethanol-producing pathways [28] in some magnitude due to lower oxygen availability. In addition, a higher agitation speed of 500 rpm had a negative effect on 2,3-BD production by the KMS005 strain. The 2,3-BD concentration and yield dramatically decreased by about 32% and 34% respectively compared to the results obtained at the agitation speed of 400 rpm. The combination between the agitation speed of 500 rpm and the aeration rate of 1.0 vvm in our study may have affected the culture environment of the broth in fermenter. It was likely that micro-aerobic conditions may be altered to partially aerobic conditions by the increase in oxygen solubility in the fermentation broth. This led the decreasing in 2,3-BD production due to an irreversible inactivation of α-acetolactate synthase by its exposure to a greater level of soluble oxygen when the agitation speed of 500 rpm was applied. However, our findings contrasted with those reported by Ma et al [6] who applied the agitation speed of 500 rpm and the aeration rate of 1.5 vvm to produce a high level of 2,3-BD from glucose within 38 h under fed-batch fermentation by *K*. *pneumoniae*.

Surprisingly, a very high concentration of maltodextrin still accumulated in the broth at 500 rpm agitation compared to those accumulated at lower agitation speeds, even though the incubation time was prolonged up to 48 h (31.3±3.1 g/L). It was likely that the KMS005 strain did not efficiently utilize maltodextrin under partially aerobic conditions reflected by high agitation rates. The explanation of this is that when culture conditions are not fully aerobic, generally combined activities of lactate dehydrogenase (*ldhA*) and α-acetolactate synthase (*budB*) play synergistic roles to dissipate pyruvate to generate lactate and 2,3-BD, thus preventing an accumulation of pyruvate and an inhibition of glycolysis caused by an imbalanced NADH/NAD^+^ ratio in the wild type strain. However, the KMS005 strain was metabolically engineered to delete *ldhA*, *adhE*, and *ackA* genes to maximize carbon flux through a 2,3-BD producing pathway [21]. Therefore, the strain only possesses a 2,3-BD producing pathway and succinate-production pathway to maintain the redox balance (NADH/NAD^+^ ratio) under fermentative conditions in a central metabolism pathway. The KMS005 strain was not able to utilize a succinate-producing pathway to maintain a redox balance of NADH/NAD^+^ due to non-restrictive anaerobic conditions and absence of an external carbon dioxide source under our fermentation conditions at 500 rpm agitation. This was confirmed by no detectable concentration of succinate in the broth. Also, at the same condition (500 rpm agitation speed), genes coding for 2,3-BD formation (*budABC* operon) may be down-regulated at the transcription level by FNR activity [9], and α-acetolactate synthase enzyme may be also partially inactivated [30] due to too much oxygen above the threshold favoring 2,3-BD production. This was observed by the decrease in concentration of 2,3-BD (Table 3). The high ratio of NADH/NAD^+^ and accumulated pyruvate usually inhibit glycerol-3-phosphate dehydrogenase enzyme thus resulting in glycolysis inhibition [30]. Therefore, the KMS005 strain stopped consuming maltodextrin to lower a glycolytic flux and to avoid a detrimental effect caused by a redox imbalance (high ratio of NADH/NAD^+^) within the cells.

Considering the carbon balance, very low amounts of by-products were detected in the fermentation broth (Table 3) and there was no observation of pyruvate in the broth. It is possible that the KMS005 strain may dissipate some of the pyruvate pool to acetyl-CoA and formate besides succinate and ethanol. Pyruvate-formate lyase (encoded by *pflB*) [30] and α-ketobutyrate/pyruvate formate-lyase (encoded by *tdcE*) [31] can assimilate pyruvate to formate and acetyl-CoA under non-fully aerobic conditions. Lots of bubbles were observed during cell growth but no formate was observed in the fermentation broth of KMS005 under our fermentation conditions. This phenomenon suggested that the strain may channel formate to produce CO_2_ and H_2_ via formate hydrogen-lyase (encoded by *fdh* and *hyc*) to support growth. Thauer et al. [36] revealed that the formate hydrogen-lyase reaction produced the electron gradient that was able to generate ATP by proton motive force. Axley et al. [37] also demonstrated that formate served as a growth substrate in many micro-organisms when carbon sources provided in the broth were limited. This effect was more pronounced where maltodextrin consumption was stalled due to the inhibition of glycolysis in the fermentation at 500 rpm agitation. Therefore, formate may be further consumed to compensate for the capability deficit in energy production due to low flux through acetate kinase (encoded by *ackA*) or low glycolytic flux in KMS005. In addition, a few amounts of acetyl-CoA were converted to acetate via a propionate kinase (TdcD) encoded by *tdcD*, which is suspected to compensate for acetate kinase activity in the KMS005 strain [13].

### Effects of substrate concentrations

Maltodextrin concentrations were varied from 5 to 25% (w/v) at the conditions of pH 6.0, aeration rate 1.0 vvm, and agitation speed 400 rpm. As shown in Table 4, maltodextrin concentration at 200 g/L provided the highest concentration and yield of 2,3-BD within 72 h. Improvement in terms of 2,3-BD concentration (74.7±0.2 g/L), productivity (1.04±0.16 g/L/ h), and yield (0.40±0.01 g/g maltodextrin) was achieved under this condition. However, employing the initial maltodextrin concentration at 250 g/L was found to affect substrate utilization. It was likely that the substrate inhibition would be considered. The comparable 2,3-BD concentration was obtained at the level of 73.4±2.4 g/L. But a dramatic reduction in gross yield (0.31±0.01 g/g maltodextrin) of about 22.5% was observed compared to that of the fermentation condition with the initial concentration of 200 g/L maltodextrin. Also, there was more sugar left over at the concentration of 78.3±2.4 g/L after 72 h incubation at the initial concentration of 250 g/L maltodextrin. Our findings are similar to the results of Wang et al. [38] who found that a significant increase of 2,3-BD concentration with an increase of cassava powder as substrate from 100 to 200 g/L in *Enterobacter cloacae*. However, when the cassava powder concentration was greater than 200 g/L, the residual sugar level sharply increased and the growth of the strain stalled. The production yield of 2,3-BD was also reduced. This may be explained by the fact that the osmotic pressure contributed from the high substrate concentrations resulted in a slower proliferation of microbial cells. The higher concentration of substrate also affected pH, viscosity and the activity of the fermentation medium. In addition, long exposure to high substrate concentration may also cause catabolic repression of microbial strains [39]. In addition, Wang et al. [38] revealed that when the initial glucose concentration was over its optimum point, not only *K*. *pneumoniae* SDM did stop the utilization of glucose substrate but the cell biomass was also significantly stalled. It had a detrimental effect on 2,3-BD production reflected by a lowering in 2,3-BD concentration compared to that obtained at the condition with optimal substrate concentration.

**Table 4:** Fermentation profile for 2,3-BD production by *K*. *oxytoca* KMS005 at various maltodextrin concentrations in AM1 medium.

Maltodextrin	Residual sugar	Max CDW	2,3-BD	Gross yield	Productivity	By-products (g/L)		
(g/L)	(g/L)	(g/L)	(g/L)	(g/g)^a^	(g/L/h)^a^	Suc	Eth	Ace
50	ND	2.8±0.1	15.1±0.3^b^^, α^	0.32±0.02^α^	0.21±0.03	1.4±0.5	1.0±0.2	2.3±0.1
100	1.6±0.1	4.8±0.1	36.6±0.6^β^	0.39±0.01^β^	0.73±0.05	2.2±0.4	0.8±0.1	1.2±0.2
150	16.6±4.1	4.8±0.3	50.4±2.4^π^	0.36±0.02^π^	1.05±0.05	1.0±0.1	0.6±0.1	0.9±0.0
200	30.7±4.5	4.9±0.0	74.7±0.2^¥^	0.40±0.01^β^	1.04±0.16	0.9±0.2	0.5±0.1	0.8±0.1
250	78.3±2.4	4.6±0.0	73.4±2.4^¥^	0.31±0.01^α^	1.02±0.03	1.1±0.4	0.6±0.1	0.7±0.3

^a^ Yield was calculated as product concentration divided by initial total sugar concentration at 48 h for substrate concentration of 50, 100 and 150 g/L and at 72 h for 200 and 250 g/L. Productivity was calculated at 48 h for substrate concentration of 50, 100 and 150 g/L and at 72h for 200 and 250 g/L.

^b^ All data represent the averages of two fermentations with standard deviations. Values bearing different Greek symbols such as “α”, “β”, “π”, and “¥” are significantly different (P < 0.05).

Noticeably, the KMS005 strain still exhibited a robustness in 2,3-BD production in AM1 medium containing high maltodextrin concentrations. The strain was able to produce up to 90 g/L of 2,3-BD from 250 g/L of maltodextrin within 120 hours incubation under batch conditions (data not shown). It seemed that the strain did not stop utilizing but gradually consumed maltodextrin, thus delaying 2,3-BD production at a high substrate concentration. *Escherichia coli* and *Klebsiella* spp. can transport linear maltodextrins via a binding protein-dependent ABC transporter consisting of maltoporin (LamB), the maltodextrin-binding protein (MBP or MalE), cytoplasmatic membrane proteins (MalF and MalG), and ATP-binding protein (MalK). The linear maltodextrins are further degraded intracellularly into glucose and glucose-1-phosphate by the enzymes amylomaltase (MalQ), maltodextrin phosphorylase (MalP), and maltodextrin glucosidase (MalZ). Among these proteins, MBP is essential for the transport of maltodextrins. MBP can bind linear maltodextrins, cyclic maltodextrins, and various maltodextrin analogues although only linear maltodextrins up to maltoheptaose are substrates for transport [40]. Maltodextrin is a polymer of glucose and is a mixture of oligosaccharide chains with different numbers of glucose sub-units connected in chains of variable length linked with α-1,4-glycosidic bonds. In our study, maltodextrin with a dextrose equivalent (DE) value of 6.4 was used. It contained only 6.4% of the reducing power of dextrose or glucose that had a DE of 100. The average degree of polymerization (DP) in maltodextrin was approximately 19 glucose sub-units (DE*DP = 120) [41]. Therefore, the transport of maltodextrin substrate used in this study was still dependent on the cleavage of the α-1,4-glycosidic linkages of high DP-maltodextrin to be low DP-maltodextrins (up to 7 glucose sub-units). In *Klebsiella* strains, the disproportionation activity of the extracellular α-cyclodextrin-glucanotransferases (CGTases) is required to degrade maltodextrin [42]. Therefore, the higher substrate concentration allowed a longer time to break down high-DP maltodextrins. Thus, the longest chain of high-DP maltodextrins may be the last one utilized and gradually consumed by the KMS005 strain resulting in delaying 2,3-BD production.

### Response Surface Methodology

Response Surface Methodology (RSM) was applied to minimize the distance of parameters to their optimum points. Box Behnken design was used to investigate the optimal levels of agitation speed, aeration rate, and substrate concentration for 2,3-BD by the KMS005 strain from maltodextrin. In RSM, upper and lower levels of significant parameters were defined based on previous results from the conventional optimization. The level of each parameter and the design matrix are shown in Table 5. However, pH was not among the selectable parameters for RSM strategy. Table 5 represents all 13 experiment runs. By applying multiple regression analysis to the experimental data, the following second-order polynomial equation was obtained:

$$\begin{array}{l} \;Y=-0.265+0.587X_{1}+0.00613X_{2}-0.00741X_{3}-0.00310X_{1}X_{2}+0.00189X_{1}X_{3}\\ +1.03*10^{5}X_{2}X_{3}+0.0647X_{1}^{2}-6.60*10^{6}X_{2}^{2}+2.45*10^{6}X_{3}^{2} \end{array}$$,

where Y is the predictable gross yield of 2,3-BD, X_1_ is the aeration rate, X_2_ is the agitation speed, and X_3_ is the maltodextrin concentration.

**Table 5:** Experimental design of RSM strategy using three independent variables.

	Parameters and their levels			2,3-BD Gross Yield^a^		
	Aeration	Agitation	Maltodextrin	Predicted	Observed	Biomass
Runs	(vvm)	(rpm)	(g/L)	(g/g)	(g/g)	(g/L)
1	0.8	350	150	0.427±0.025	0.438±0.020	4.4±0.2
2	1	250	250	0.149±0.025	0.203±0.020	3.2±0.3
3	1.2	350	150	0.393±0.025	0.430±0.010	4.6±0.1
4	1	350	200	0.354±0.025	0.355±0.016	4.2±0.3
5	1.2	350	250	0.337±0.025	0.348±0.014	4.5±0.1
6	1	250	150	0.345±0.025	0.364±0.006	4.1±0.1
7	1	450	250	0.347±0.025	0.341±0.002	4.6±0.1
8	1.2	450	200	0.279±0.025	0.263±0.004	4.8±0.3
9	0.8	350	250	0.296±0.025	0.277±0.008	3.9±0.3
10	0.8	250	200	0.180±0.025	0.216±0.001	3.4±0.2
11	1	450	150	0.337±0.025	0.306±0.005	4.9±0.1
12	1.2	250	200	0.307±0.025	0.302±0.011	3.8±0.3
13	0.8	450	200	0.399±0.025	0.431±0.001	4.9±0.2

^a^ Gross yield was calculated at 72 h.

From a model analysis (S2 Table), the Model F-value of 23.61 implied the significance of the model. Values of Prob > F less than 0.0001 indicated the model terms are significant. In this case X_1_, X_3_, X_1_X_2_, X_2_X_3_, and X_2_^2^ were significant model terms (P < 0.0001). P value above 0.0001 suggested that X_2_, X_1_X_3_, X_1_^2^, and X_3_^2^ were not significant. The fitness of the model was checked by the coefficient of determination R^2^, which was calculated to be 0.9341 showing that 93.41% of variability in the response could be explained by the model. An R^2^ value higher than 0.9 was considered to have a very high correlation. The value of the adjusted determination coefficient (Adj R^2^ = 89.45%) was also satisfied to advocate for a high significance of the model. The predicted R^2^ of 0.8084 was in reasonable agreement with the Adj R^2^ of 0.8945. The Adeq Precision measures the signal to noise ratio that should be greater than 4 [43]. Our ratio of 16.228 indicated an adequate and desirable signal. Therefore, this model can be used for navigating the design space. The regression model is also reasonable to analyze the trends in the responses.

**Fig 2:**
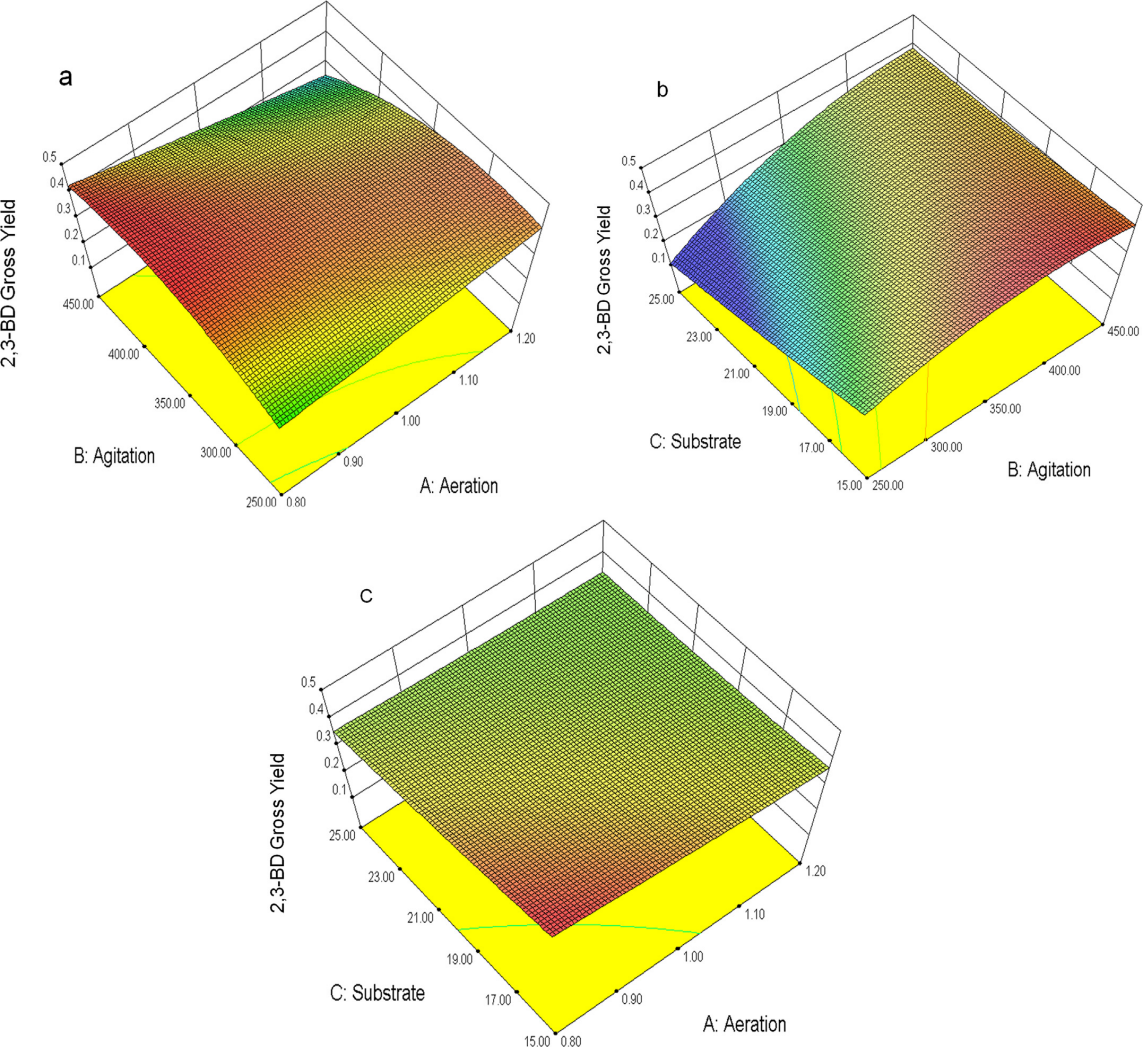
**3D structures of optimum points of aeration, agitation, and substrate concentration:** (a) the plot between aeration and agitation, (b) the plot between agitation and substrate concentration, (c) the plot between aeration and substrate concentration.

Furthermore, the effects of aeration, agitation, and maltodextrin concentration on 2,3-BD production were also evaluated by the 3D response surface (Fig 2A–2C). The red surfaces in 3D structures corresponded to maximum responses of 2,3-BD concentration. Based on the model and the 3D plots, the optimal levels of aeration, agitation, and maltodextrin concentration were 0.8 vvm, 400 rpm and 15% (w/v) respectively. The predicted maximum 2,3-BD yield was calculated at the level of 0.45±0.025 g/g maltodextrin. From the model, all three significant parameters provided an interaction effect on each other. Agitation speed had a great significant effect on the model. It also had interaction effects on the other two parameters, aeration rate and substrate concentration, to drive flow and mixing as well as optimum micro-aerobic conditions for 2,3-BD production yield in the KMS005 strain. Aeration rate and substrate concentration had no significance on their main effects or on their interaction with each other. Both of them had significance on the model by means of their interactions with agitation speed. This may imply that the difference in flow of mixing caused by different agitation speeds and aeration rates reflected cell growth and 2,3-BD production yield by the KMS005 strain. Lee et al. [7] claimed that aeration rate (3.5 vvm) and CLS concentration (45 mL/L) did not significantly affect the proportion of 2,3-BD. This supported our findings that no interaction effect between aeration rates (X_1_) and substrate concentration (X_3_) was found. Fortunately, our results found that the optimized aeration rate at 0.8 vvm fell in the range of 0.5–1.5 vvm. The rate is expected to be easily maintained in scaling up [27]. The optimized substrate level at 15% (w/v) was estimated to be equivalent to glucose concentration of 140 g/L. The concentration was appropriate to fermentation under batch condition, as suggested by Yu and Saddler [5] who stated that final 2,3-BD concentrations were found to be highest for cultures grown on the initial glucose concentration of 150 g/L, particularly when the inoculum was first acclimatized to high sugar levels.

### Validation of the mathematic model obtained from RSM strategy

**Fig 3:**
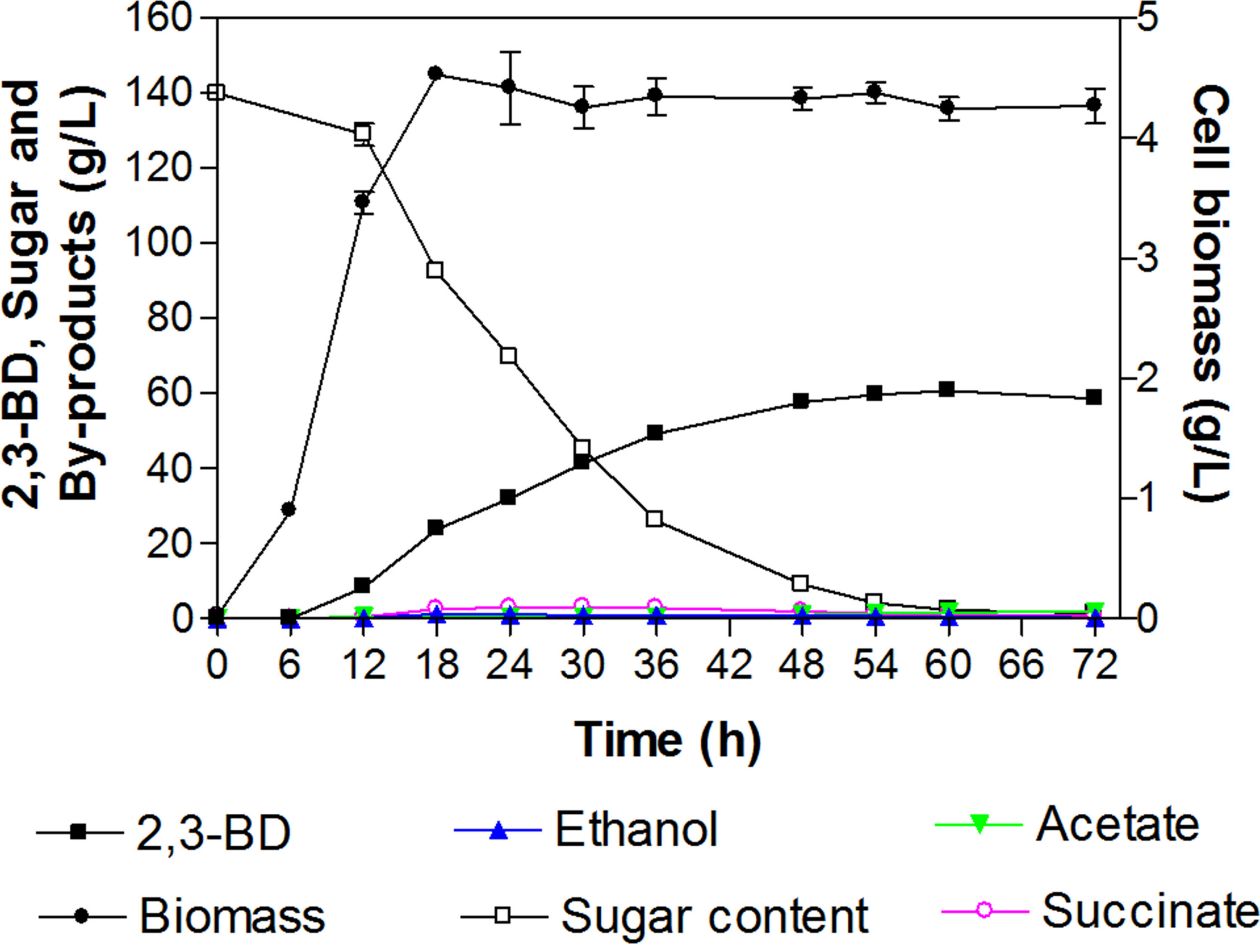
Batch fermentation profile for 2,3-BD production from maltodextrin performed under optimized agitation speed, aeration rate, and maltodextrin concentration.

**Fig 4:**
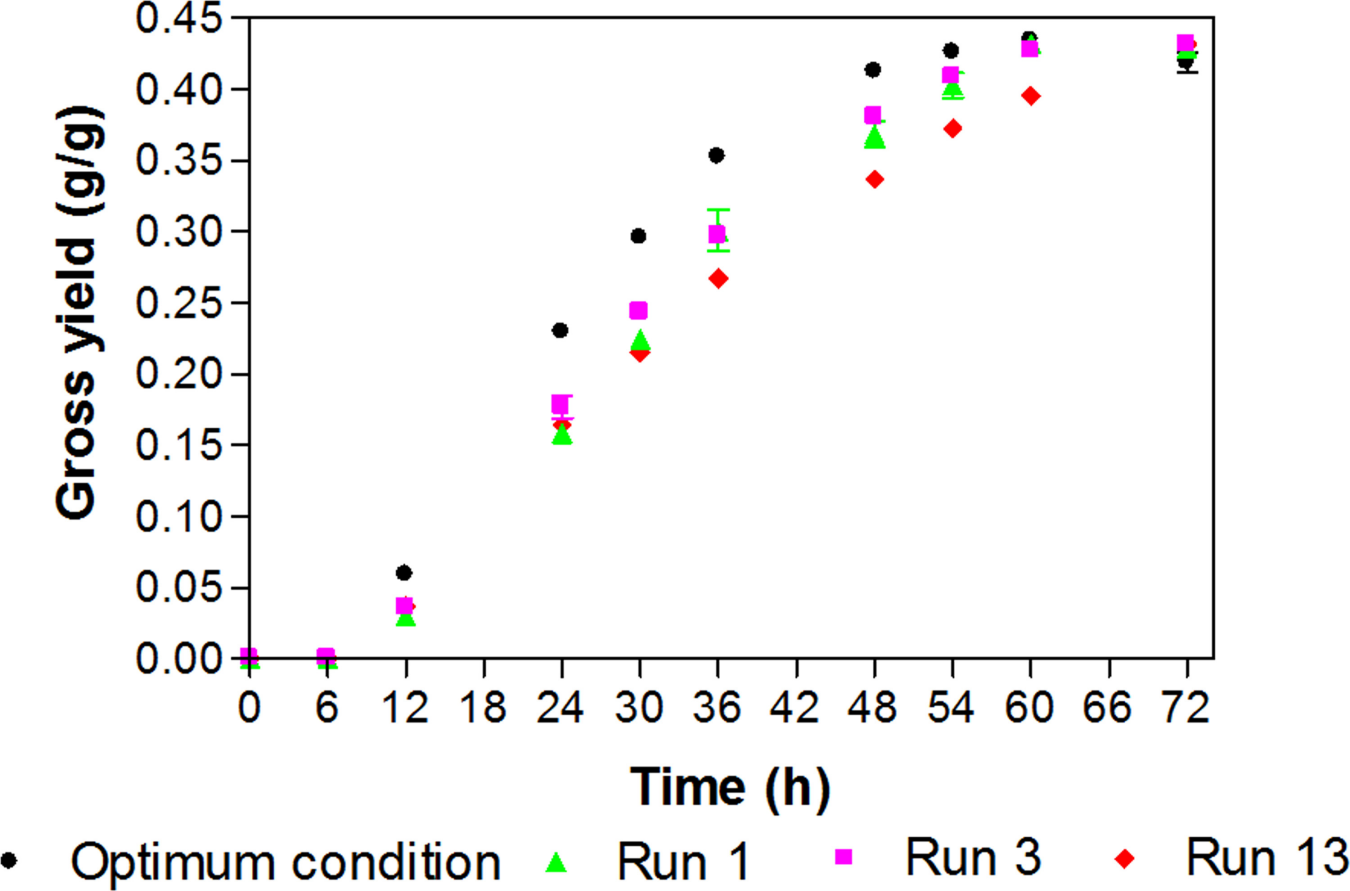
Gross yield comparisons obtained during fermentation.

To confirm the availability of the mathematic equation for predicting maximum 2,3-BD production yield, the experiment was carried out in batch fermentation in a 2 L fermenter to validate the model. Optimum 2,3-BD conditions were observed by the model at the agitation speed of 400 rpm, and aeration rate of 0.8 vvm with 150 g/L of maltodextrin. At 60 h incubation, 2,3-BD production at 0.433±0.002 was obtained (Fig 3; S3 Table). The validated yield of 2,3-BD obtained from the condition using optimized parameters was statistically acceptable and satisfactory compared to that of the predicted yield at 0.450±0.025 g/g maltodextrin calculated by the model. Interestingly, the optimum condition was able to achieve the yield of 0.433 g/g maltodextrin at a shorter incubation time (60 h) than that of other conditions (obtained at 72 h) (Table 5). Also, 2,3-BD production yield from optimized conditions was elucidated to be the best among other conditions, especially those from run 1, 3, and 13 (Fig 4; S4 Table).

Unlike the conventional method, RSM strategy provided advantages for fermentative 2,3-BD production. The aeration rate was reduced from 1.0 to 0.8 vvm thus resulting in lowering the energy consumption for 2,3-BD production. Second, the production of 2,3-BD by the KMS005 strain from 150 g/L maltodextrin (48 h) in terms of concentration (50.4±2.4 versus 57.7±0.5 g/L), yield (0.360±0.017 versus 0.412±0.003 g/g) productivity (1.05±0.05 versus 1.20±0.01 g/L/h) was significantly improved (Table 4 and Fig 3).

### Fed-batch fermentation

**Fig 5:**
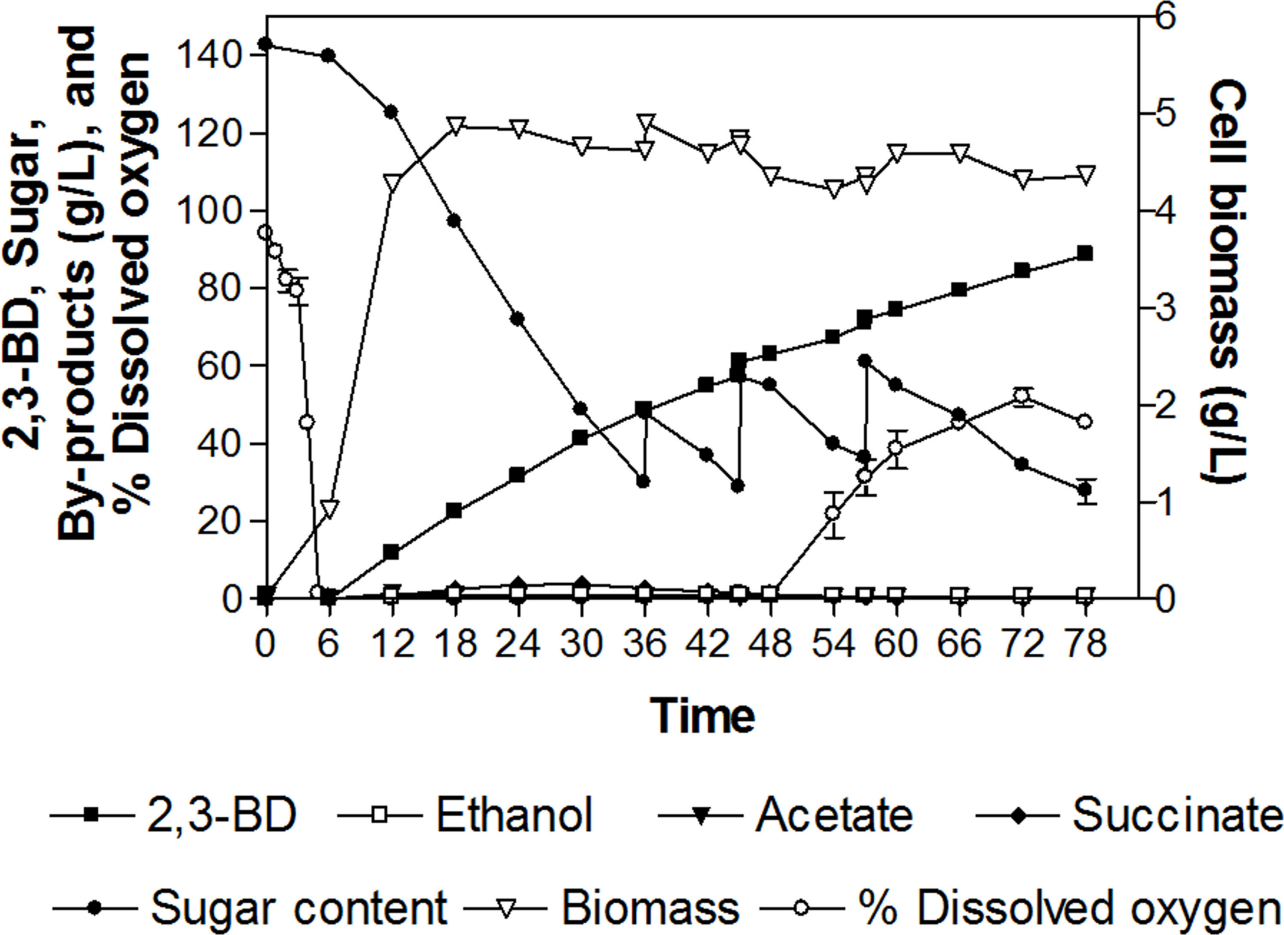
Fed-batch fermentation profile for 2,3-BD production from maltodextrin by KMS005 under optimum conditions.

The optimum condition in RSM was applied in the interim fed-batch fermentation. At 78 h incubation, 2,3-BD concentration, gross yield, and productivity were achieved at 88.1±0.2 g/L, 0.412±0.001 g/g maltodextrin, and 1.13±0.01 g/L/h respectively. The by-products including succinate, ethanol, and acetate were at concentrations of 0.3±0.1, 0.5±0.1, and 0.8±0.2 g/L respectively (Fig 5; S5 Table). The concentrations of by-products under the fed-batch experiment were less than those obtained under batch fermentation. Considering dissolved oxygen, cells consumed oxygen until 48 h before the dissolved oxygen increased gradually until the end of fermentation. Since 2,3-BD became a mixed growth-associated product by the KMS005 strain, its incremental production of 2,3-BD concomitantly occurred in the log phase addition to the stationary phase. In wide type strains, all enzymes in 2,3-BD pathway are activated in the late log and stationary phases under oxygen limitation, and induced by acetate at low pH [44]. Recently, Wong et al. [45] stated that 2,3-BD production by *Klebsiella* Zmd30 was also found to be growth-associated provoking lower productivity in fed-batch than that obtained in batch mode. This supported our findings which slightly decrease by 1.13 g/L/h of fed batch productivity was obtained.

Our study was the first to report the use of maltodextrin as a cheap carbon substrate for high production yield of 2,3-BD. Even though the titer and productivity of 2,3-BD in this study was not be the highest level ever published (Table 6) due to the use of mineral salt medium that contained the least nutrients essential for bacterial growth. However, most previously published works on 2,3-BD production were performed in media composed of complex, rich, and expensive nutrients. Antibiotics were also supplied to maintain heterologous gene expression for activating 2,3-BD producing pathway [6,18,35]. These led to high production of 2,3-BD in terms of titers and productivities, but they contributed to an increase in production costs including nutrients and chemical prices, and required additional steps of product recovery. However, simple mineral salt medium with less nitrogen sources and trace metals, and transparently-pure maltodextrin used in our study are expected to reduce some obstacles in product recovery. Consequently, costs related to medium preparation and waste disposal also decreased.

**Table 6:** Comparison of 2,3-BD production by *Klebsiella* species from glucose-based medium and microorganisms.

Organism	Substrate/Medium/Condition	2,3-BD	Yield	Productivity	References
		(g/L)	(g/g)	(g/L/h)	
*K*. *oxytoca* KMS005	150 g/L maltodextrin (140 g/L of glucose), supplemented with AM1 mineral salt medium, pH6.0, 0.8 vvm, Fed batch, 78 h incubation, 37°C, 400 rpm.	88.1	0.412	1.13 ^a^	This study
*K*. *oxytoca* M1/pUC18CM-*budC*	80–100 g/L glucose, supplemented with define medium, 5 g/L yeast extract 10 g/L casamino acid, 25 μg/ mL chloramphenicol, pH6.0, 1.0 vvm, Fed batch, 97 h incubation time, 30°C, 400 rpm.	142.5	0.42	1.47 ^a^	[34]
*K*. *oxytoca* NBRF4 (chemical mutation)	44 g/L glucose, supplemented with YP medium, pH 4.3, aeration 10% dissolved oxygen, batch, 18 h incubation, 38°C, 200 rpm.	14.4	0.32	0.78	[47]
*K*. *oxytoca*	90 g/L glucose, supplemented with a medium containing 5 g/L yeast extract, trace elements, pH 6.5, 1.0 vvm, batch, 30 h incubation, 37°C without shaking.	30	0.33	1.15	[15]
*K*. *oxytoca* ACCC 10370	Corncob hemicellulose hydrolysate (39.5 g/L xylose, 9.9 g/L glucose, 1.5 g/L arabinose, and 1.8 g/L acetate), supplemented with a medium containing 1.5 g/L yeast extract, pH 6, 0.3 vvm, batch, 48 h incubation, 37°C, 300 rpm.	23.5	0.46	0.49	[48]
*K*. *oxytoca* ME-UD-3 (Δ*ald*A)	200 g/L glucose, supplemented with a medium containing EDTA 0.05 M, pH 6.5, 1.0 vvm, fed-batch, 60 h incubation, 37°C, 200 rpm.	130	0.48	1.63 ^a^	[49]
*K*. *oxytoca* ME-UD-3	220 g/L glucose, supplemented with a medium, pH 6.0, aeration 1.0 vvm, batch, 81 h incubation, 37°C, 200 rpm.	86.2	0.39	1.06	[16]
*K*. *pneumoniae* SDM isolated from soil	Total glucose utilized 168 g/L, supplemented with medium, pH 7.0, 1.5 vvm, fed-batch, 48 h incubation time, 37°C, 500 rpm.	150	0.43	4.21^a^	[6]

^a^ Productivity calculated under fed-batch.

In addition, bio-based 2,3-BD production appears economically attractive because the estimated fermentation cost was about $2.04/kg 2,3-BD produced, based on the results in our study. The cost included AM1 medium at $0.43/kg and maltodextrin at $0.69/kg. As the market price of glucose is about $1.0–1.50/kg, the fermentation cost for 2,3-BD production from maltodextrin would be cheaper than that derived from glucose. In addition, it is likely that the price of maltodextrin derived from cassava starch was cheap when no enzyme utilization in hydrolysis and pre-treatment step of the raw material were required in our processes. Unlike maltodextrin, the price of cassava starch is sold in the market at $0.40/kg, much cheaper than maltodextrin [21]. However, high investment cost on enzymatic hydrolysis is required for saccharification steps. The prices of amyloglucosidase and α-amylase at costs of $7.57/kg and $3.50/kg [21] respectively would contribute to higher production cost of 2,3-BD if cassava starch was utilized. Thailand is known as one of the world’s leading countries for production and export of cassava starch (about 2 million tons annually). The use of cassava starch in the form of maltodextrin for 2,3-BD production is not concerned to be competitive for human consumption [14, 46]. Compared to 2,3 BD production derived from chemical synthesis, the selling price in the market ranged from $9.12 to $19.77/kg [17]. Therefore, our 2,3-BD fermentation cost at $2.04/kg is a solid benefit and makes it possible to bring the model development into industrial production. Further improvement of 2,3-BD production in terms of concentration and productivity is yet possible by means of the KMS005 strain development.

## Conclusion

To the best of our knowledge, this is the first study on 2,3-BD production from maltodextrin, an abundant and pure substrate derived from cassava. Fermentative operational parameters including pH, aeration, agitation, and substrate concentration were optimized. Strain *K*. *oxytoca* KMS005 is able to efficiently hydrolyze maltodextrin and produce 2,3-BD at the concentration and yield of 88.1 g/L and 0.412 g/g along with minor amounts of by-products within 78 h under optimum conditions in fed-batch mode. Meanwhile, the fermentation process was performed considering the reduction of the total production cost by lowering the cost in fermentation and purification steps by employing simple mineral salt medium without additional complex nutrients, utilization of pure and abundant substrate derived from cassava rather than directly refined glucose, eliminating the enzymatic hydrolysis step, and taking advantages of using bio-catalyst downstream processing as less by-products formation. However, further study is required to improve titer, yield, and productivity to meet the commercial requirements of 2,3-BD production in the near future.

## Supporting Information

S1 Table Data for cell biomass and 2,3-BD production at different agitation speeds of 300 and 400 rpm. (DOC)

S2 Table ANOVA summary for model analysis (DOC)

S3 Table Data for fermentative products (g/L) during 2,3-BD production by the KMS005 strain in batch mode using maltodextrin as substrate under the optimized condition. (DOC)

S4 Table Data for gross yield between runs in RSM experiments. (DOC)

S5 Table Data for fermentative products (g/L) and dissolve oxygen (%) during 2,3-BD production in fed-batch mode using maltodextrin as substrate under the optimized condition. (DOC)
