# Supplementary material for: Process Optimization on Micro-Aeration Supply for High Production Yield of 2,3-Butanediol from Maltodextrin by Metabolically-Engineered Klebsiella oxytoca
Source: PLoS One. 2016 Sep 7;11(9):e0161503. doi: 10.1371/journal.pone.0161503 (PMC5014425; doi:10.1371/journal.pone.0161503)
Supplement: S1 Table — (DOC) [file pone.0161503.s001.doc]

**S1 Table. Data for cell biomass and 2,3-BD production at different agitation speeds of**

**300 and 400 rpm.**

| **Time**  **(h)** | **300 rpm**  **(Biomass, g/L)** | | | **400 rpm**  **(Biomass, g/L)** | | | **300 rpm**  **(2,3-BD, g/L)** | | | **400 rpm**  **(2,3-BD, g/L)** | | |
| --- | --- | --- | --- | --- | --- | --- | --- | --- | --- | --- | --- | --- |
|  | **1** | **2** | **3** | **1** | **2** | **3** | **1** | **2** | **3** | **1** | **2** | **3** |
| 0 | 0.03 | 0.03 | 0.03 | 0.03 | 0.03 | 0.03 | 0 | 0 | 0 | 0 | 0 | 0 |
| 12 | 2.36 | 2.37 | 2.36 | 3.73 | 3.63 | 3.68 | 5.47 | 5.10 | 5.31 | 9.54 | 9.35 | 9.44 |
| 24 | 4.11 | 4.05 | 4.08 | 4.25 | 4.18 | 4.21 | 23.19 | 22.64 | 23.25 | 26.53 | 28.92 | 27.56 |
| 36 | 4.68 | 4.52 | 4.63 | 4.10 | 4.39 | 4.24 | 34.58 | 35.55 | 33.92 | 33.08 | 35.92 | 34.95 |
| 48 | 4.39 | 4.28 | 4.17 | 4.17 | 4.14 | 4.20 | 36.19 | 37.08 | 36.77 | 33.51 | 37.19 | 35.12 |
