# Supplementary material for: Process Optimization on Micro-Aeration Supply for High Production Yield of 2,3-Butanediol from Maltodextrin by Metabolically-Engineered Klebsiella oxytoca
Source: PLoS One. 2016 Sep 7;11(9):e0161503. doi: 10.1371/journal.pone.0161503 (PMC5014425; doi:10.1371/journal.pone.0161503)
Supplement: S2 Table — (DOC) [file pone.0161503.s002.doc]

**S2 Table. ANOVA summary for model analysis**

| ANOVA for Response Surface Quadratic Model | | | | |  |  |  |
| --- | --- | --- | --- | --- | --- | --- | --- |
| Analysis of variance table [Partial sum of squares - Type III] | | | | | |  |  |
|  | Sum of |  | Mean | F | p-value |  | |
| Source | Squares | Df | Square | Value | Prob > F |  | |
| Block | 1.66E-06 | 1 | 1.66E-06 |  |  |  | |
| Model | 0.133641 | 9 | 0.014849 | 23.61415818 | < 0.0001 | significant | |
| X1-  Aeration | 0.00016 | 1 | 0.00016 | 0.253662887 | 0.6218 |  | |
| X2-Agitation | 0.018301 | 1 | 0.018301 | 29.1043915 | < 0.0001 |  | |
| X3-Substrate | 0.03458 | 1 | 0.03458 | 54.99161255 | < 0.0001 |  | |
| X1X2 | 0.030785 | 1 | 0.030785 | 48.95692557 | < 0.0001 |  | |
| X1X3 | 0.002496 | 1 | 0.002496 | 3.969613577 | 0.0649 |  | |
| X2X4 | 0.018489 | 1 | 0.018489 | 29.40303056 | < 0.0001 |  | |
| X1­­­­2 | 3.06E-05 | 1 | 3.06E-05 | 0.048640668 | 0.8284 |  | |
| X22 | 0.019856 | 1 | 0.019856 | 31.57723914 | < 0.0001 |  | |
| X32 | 0.000131 | 1 | 0.000131 | 0.207642375 | 0.6551 |  | |
| Residual | 0.009432 | 15 | 0.000629 |  |  |  | |
| Core Total | 0.143075 | 25 |  |  |  |  |  |

Std. Dev=0.025, C.V. %=7.55, R2=0.9341, Adj R2=0.8945, Pred R2=0.8084, Adeq Precision=16.228.
