# Supplementary material for: Process Optimization on Micro-Aeration Supply for High Production Yield of 2,3-Butanediol from Maltodextrin by Metabolically-Engineered Klebsiella oxytoca
Source: PLoS One. 2016 Sep 7;11(9):e0161503. doi: 10.1371/journal.pone.0161503 (PMC5014425; doi:10.1371/journal.pone.0161503)
Supplement: S3 Table — (DOC) [file pone.0161503.s003.doc]

**S3 Table. Data for fermentative products (g/L) during 2,3-BD production by KMS005 strain in batch mode using maltodextrin as**

**substrate under the optimized condition.**

| **Time (h)** | **2,3-BD** | | | **Ethanol** | | | **Acetate** | | | **Succinate** | | | **Cell biomass** | | | **Sugar content** | | |
| --- | --- | --- | --- | --- | --- | --- | --- | --- | --- | --- | --- | --- | --- | --- | --- | --- | --- | --- |
|  | **1** | **2** | **3** | **1** | **2** | **3** | **1** | **2** | **3** | **1** | **2** | **3** | **1** | **2** | **3** | **1** | **2** | **3** |
| 0 | 0 | 0 | 0 | 0 | 0 | 0 | 0 | 0 | 0 | 0 | 0 | 0 | 0.03 | 0.03 | 0 | 139.97 | 140.01 | 140.11 |
| 6 | 0 | 0 | 0 | 0 | 0 | 0 | 0 | 0 | 0 | 0 | 0 | 0 | 0.93 | 0.86 | 0 | 135.27 | 137.65 | 136.11 |
| 12 | 7.41 | 9.16 | 8.87 | 0 | 0.46 | 0.11 | 0.56 | 0.76 | 0.63 | 0.38 | 0.62 | 0.54 | 3.37 | 3.55 | 3.44 | 125.97 | 131.94 | 127.55 |
| 18 | 23.05 | 23.75 | 21.33 | 0.95 | 1.20 | 1.35 | 0.53 | 0.22 | 0.44 | 2.45 | 2.57 | 2.52 | 4.51 | 4.55 | 4.52 | 87.26 | 92.29 | 90.53 |
| 24 | 31.44 | 32.73 | 33.01 | 1.01 | 1.25 | 1.11 | 0.45 | 0.13 | 0.36 | 2.68 | 3.24 | 2.85 | 4.12 | 4.71 | 4.41 | 70.54 | 68.91 | 71.25 |
| 30 | 41.41 | 41.33 | 41.89 | 1.06 | 1.02 | 1.21 | 0.51 | 0.09 | 0.88 | 2.89 | 3.31 | 3.04 | 4.08 | 4.42 | 4.25 | 46.84 | 43.79 | 44.36 |
| 36 | 49.22 | 49.42 | 48.59 | 0.98 | 1.06 | 0.84 | 0.25 | 0.34 | 0.25 | 2.55 | 3.11 | 2.89 | 4.19 | 4.49 | 4.32 | 27.18 | 24.83 | 25.11 |
| 48 | 58.07 | 57.34 | 57.88 | 0.94 | 0.83 | 1.02 | 1.11 | 0.79 | 1.21 | 2.11 | 1.62 | 1.95 | 4.23 | 4.42 | 4.31 | 9.73 | 8.45 | 8.55 |
| 54 | 60.22 | 59.01 | 61.14 | 0.84 | 0.74 | 0.77 | 1.60 | 1.15 | 1.44 | 1.60 | 1.14 | 1.33 | 4.29 | 4.46 | 4.35 | 4.21 | 4.18 | 4.32 |
| 60 | 60.97 | 60.51 | 62.01 | 0.63 | 0.29 | 0.47 | 1.76 | 1.64 | 1.85 | 1.11 | 0.84 | 0.92 | 4.33 | 4.14 | 4.21 | 2.62 | 2.05 | 2.55 |
| 72 | 59.61 | 57.55 | 58.46 | 0.48 | 0.25 | 0.54 | 2.03 | 1.86 | 1.64 | 0.33 | 1.17 | 0.55 | 4.41 | 4.12 | 4.23 | 1.53 | 1.42 | 1.39 |
