# Supplementary material for: Process Optimization on Micro-Aeration Supply for High Production Yield of 2,3-Butanediol from Maltodextrin by Metabolically-Engineered Klebsiella oxytoca
Source: PLoS One. 2016 Sep 7;11(9):e0161503. doi: 10.1371/journal.pone.0161503 (PMC5014425; doi:10.1371/journal.pone.0161503)
Supplement: S4 Table — (DOC) [file pone.0161503.s004.doc]

**S4 Table. Data for gross yield between runs in RSM experiments.**

| **Time (h)** | **Optimum** | | | **Run 1** | | | **Run 3** | | | **Run 13** | | |
| --- | --- | --- | --- | --- | --- | --- | --- | --- | --- | --- | --- | --- |
|  | **1** | **2** | **3** | **1** | **2** | **3** | **1** | **2** | **3** | **1** | **2** | **3** |
| 0 | 0 | 0 | 0 | 0 | 0 | 0 | 0 | 0 | 0 | 0 | 0 | 0 |
| 6 | 0 | 0 | 0 | 0 | 0 | 0 | 0 | 0 | 0 | 0 | 0 | 0 |
| 12 | 0.052 | 0.065 | 0.058 | 0.024 | 0.035 | 0.029 | 0.033 | 0.037 | 0.035 | 0.036 | 0.031 | 0.034 |
| 24 | 0.224 | 0.233 | 0.285 | 0.153 | 0.162 | 0.158 | 0.169 | 0.184 | 0.177 | 0.164 | 0.156 | 0.141 |
| 30 | 0.295 | 0.295 | 0.295 | 0.217 | 0.231 | 0.224 | 0.237 | 0.249 | 0.241 | 0.215 | 0.204 | 0.211 |
| 36 | 0.351 | 0.353 | 0.354 | 0.315 | 0.286 | 0.306 | 0.291 | 0.302 | 0.311 | 0.266 | 0.255 | 0.274 |
| 48 | 0.414 | 0.409 | 0.412 | 0.358 | 0.377 | 0.366 | 0.377 | 0.383 | 0.380 | 0.336 | 0.342 | 0.348 |
| 54 | 0.430 | 0.421 | 0.425 | 0.394 | 0.411 | 0.401 | 0.409 | 0.407 | 0.401 | 0.372 | 0.375 | 0.377 |
| 60 | 0.435 | 0.432 | 0.434 | 0.428 | 0.434 | 0.430 | 0.428 | 0.424 | 0.425 | 0.395 | 0.391 | 0.389 |
| 72 | 0.425 | 0.411 | 0.419 | 0.428 | 0.428 | 0.423 | 0.431 | 0.431 | 0.439 | 0.431 | 0.429 | 0.433 |
