# Supplementary material for: Process Optimization on Micro-Aeration Supply for High Production Yield of 2,3-Butanediol from Maltodextrin by Metabolically-Engineered Klebsiella oxytoca
Source: PLoS One. 2016 Sep 7;11(9):e0161503. doi: 10.1371/journal.pone.0161503 (PMC5014425; doi:10.1371/journal.pone.0161503)
Supplement: S5 Table — (DOC) [file pone.0161503.s005.doc]

**S5 Table.** **Data for fermentative products (g/L) and dissolve oxygen (%) during 2,3-BD production in fed-batch mode**

**using maltodextrin as substrate under the optimized condition.**

| **Time (h)** | **2,3-BD** | | | **Ethanol** | | | **Acetate** | | | **Succinate** | | | **Sugar** | | | **Biomass** | | | **% Dissolved oxygen** | | |
| --- | --- | --- | --- | --- | --- | --- | --- | --- | --- | --- | --- | --- | --- | --- | --- | --- | --- | --- | --- | --- | --- |
|  | **1** | **2** | **3** | **1** | **2** | **3** | **1** | **2** | **3** | **1** | **2** | **3** | **1** | **2** | **3** | **1** | **2** | **3** | **1** | **2** | **3** |
| 0 | 0 | 0 | 0 | 0 | 0 | 0 | 0 | 0 | 0 | 0 | 0 | 0 | 142 | 143 | 143 | 0.03 | 0.03 | 0.03 | 95 | 93 | 95 |
| 1 |  |  |  |  |  |  |  |  |  |  |  |  |  |  |  |  |  |  | 90.7 | 88 | 92.1 |
| 2 |  |  |  |  |  |  |  |  |  |  |  |  |  |  |  |  |  |  | 84.8 | 79.2 | 86.3 |
| 3 |  |  |  |  |  |  |  |  |  |  |  |  |  |  |  |  |  |  | 75.8 | 82.6 | 77.1 |
| 4 |  |  |  |  |  |  |  |  |  |  |  |  |  |  |  |  |  |  | 43.8 | 46.5 | 42.5 |
| 5 |  |  |  |  |  |  |  |  |  |  |  |  |  |  |  |  |  |  | 0.8 | 1.8 | 0.5 |
| 6 | 0 | 0 | 0 | 0 | 0 | 0 | 0 | 0 | 0 | 0 | 0 | 0 | 139 | 140 | 137 | 0.91 | 1.14 | 1.08 | 0 | 0.8 | 0.2 |
| 12 | 11.86 | 12.02 | 11.91 | 0.49 | 0.55 | 0.52 | 1.49 | 1.52 | 1.55 | 0.76 | 0.85 | 0.61 | 125.10 | 121.56 | 123.11 | 4.27 | 4.14 | 4.22 | 0.1 | 0 | 0 |
| 18 | 22.43 | 23.01 | 22.77 | 0.93 | 0.84 | 1.05 | 1.13 | 1.16 | 1.12 | 2.60 | 2.67 | 2.44 | 97.10 | 95.85 | 96.25 | 4.86 | 4.59 | 4.61 | 0.1 | 0 | 0 |
| 24 | 31.58 | 32.29 | 31.85 | 0.99 | 0.98 | 1.11 | 0.80 | 1.42 | 0.98 | 3.73 | 3.78 | 3.12 | 71.80 | 72.09 | 71.92 | 4.83 | 4.75 | 4.77 | 0.1 | 0.2 | 0 |
| 30 | 41.18 | 40.31 | 40.98 | 1.08 | 1.14 | 1.21 | 1.17 | 1.21 | 1.29 | 3.76 | 3.85 | 3.41 | 48.52 | 46.28 | 47.55 | 4.65 | 4.52 | 4.59 | 0.1 | 0.5 | 0.3 |
| 36 | 48.63 | 46.84 | 47.26 | 0.98 | 1.31 | 1.22 | 0.52 | 0.59 | 0.55 | 2.86 | 2.71 | 2.98 | 29.97 | 28.91 | 27.32 | 4.61 | 4.57 | 4.59 | 0.4 | 0.4 | 0.4 |
| 42 | 54.79 | 56.12 | 55.48 | 0.86 | 0.79 | 0.81 | 0.47 | 0.51 | 0.50 | 2.09 | 2.15 | 2.26 | 36.84 | 39.58 | 38.41 | 4.58 | 4.51 | 4.55 | 0.1 | 1 | 0.5 |
| 45 | 57.43 | 56.69 | 56.99 | 0.77 | 0.75 | 0.77 | 0.47 | 0.43 | 0.45 | 1.55 | 1.62 | 1.71 | 28.83 | 26.59 | 28.11 | 4.73 | 4.81 | 4.71 | 0.7 | 2.1 | 1.4 |
| 48 | 63.07 | 65.98 | 64.10 | 0.80 | 0.76 | 0.91 | 0.51 | 0.46 | 0.48 | 1.24 | 1.28 | 1.29 | 54.77 | 57.12 | 55.65 | 4.35 | 4.24 | 4.30 | 1.3 | 1.2 | 1.1 |
| 54 | 67.18 | 66.37 | 67.92 | 0.70 | 0.71 | 0.81 | 0.51 | 0.51 | 0.49 | 0.64 | 0.67 | 0.55 | 39.78 | 35.12 | 37.14 | 4.21 | 4.26 | 4.25 | 27.5 | 15.8 | 22.3 |
| 57 | 71.12 | 70.54 | 70.91 | 0.73 | 0.62 | 0.61 | 0.56 | 0.48 | 0.52 | 0.50 | 0.53 | 0.41 | 36.29 | 35.87 | 35.98 | 4.35 | 4.20 | 4.28 | 35.9 | 26.7 | 30.2 |
| 60 | 74.45 | 74.42 | 74.26 | 0.66 | 0.62 | 0.64 | 0.54 | 0.61 | 0.59 | 0.33 | 0.35 | 0.41 | 54.78 | 55.01 | 56.23 | 4.58 | 4.34 | 4.41 | 43.4 | 33.6 | 38.6 |
| 66 | 79.26 | 78.50 | 78.98 | 0.55 | 0.51 | 0.52 | 0.61 | 0.62 | 0.59 | 0.23 | 0.20 | 0.29 | 47.12 | 48.36 | 47.56 | 4.58 | 4.49 | 4.45 | 46.2 | 44.1 | 45.1 |
| 72 | 84.13 | 84.14 | 83.22 | 0.61 | 0.54 | 0.54 | 0.64 | 0.55 | 0.77 | 0.18 | 0.25 | 0.20 | 34.36 | 35.80 | 34.89 | 4.31 | 4.38 | 4.32 | 54.3 | 49.7 | 52.7 |
| 78 | 88.87 | 88.26 | 87.11 | 0.49 | 0.57 | 0.61 | 0.71 | 0.74 | 0.98 | 0.18 | 0.26 | 0.22 | 24.58 | 30.97 | 28.14 | 4.36 | 4.40 | 4.35 | 44.2 | 46.5 | 45.1 |
